# Transcription Factor SP4 Is a Susceptibility Gene for Bipolar Disorder

**DOI:** 10.1371/journal.pone.0005196

**Published:** 2009-04-09

**Authors:** Xianjin Zhou, Wei Tang, Tiffany A. Greenwood, Shengzhen Guo, Lin He, Mark A. Geyer, John R. Kelsoe

**Affiliations:** 1 Department of Psychiatry, University of California San Diego, La Jolla, California, United States of America; 2 Department of Psychiatry, VA San Diego Healthcare System, La Jolla, California, United States of America; 3 Bio-X Life Science Research Center, Shanghai Jiaotong University, Shanghai, People's Republic of China; Centre National de la Recherche Scientifique, France

## Abstract

The Sp4 transcription factor plays a critical role for both development and function of mouse hippocampus. Reduced expression of the mouse *Sp4* gene results in a variety of behavioral abnormalities relevant to human psychiatric disorders. The human SP4 gene is therefore examined for its association with both bipolar disorder and schizophrenia in European Caucasian and Chinese populations respectively. Out of ten SNPs selected from human SP4 genomic locus, four displayed significant association with bipolar disorder in European Caucasian families (rs12668354, p = 0.022; rs12673091, p = 0.0005; rs3735440, p = 0.019; rs11974306, p = 0.018). To replicate the genetic association, the same set of SNPs was examined in a Chinese bipolar case control sample. Four SNPs displayed significant association (rs40245, p = 0.009; rs12673091, p = 0.002; rs1018954, p = 0.001; rs3735440, p = 0.029), and two of them (rs12673091, rs3735440) were shared with positive SNPs from European Caucasian families. Considering the genetic overlap between bipolar disorder and schizophrenia, we extended our studies in Chinese trios families for schizophrenia. The SNP7 (rs12673091, p = 0.012) also displayed a significant association. The SNP7 (rs12673091) was therefore significantly associated in all three samples, and shared the same susceptibility allele (A) across all three samples. On the other hand, we found a gene dosage effect for mouse Sp4 gene in the modulation of sensorimotor gating, a putative endophenotype for both schizophrenia and bipolar disorder. The deficient sensorimotor gating in *Sp4* hypomorphic mice was partially reversed by the administration of dopamine D2 antagonist or mood stabilizers. Both human genetic and mouse pharmacogenetic studies support Sp4 gene as a susceptibility gene for bipolar disorder or schizophrenia. The studies on the role of Sp4 gene in hippocampal development may provide novel insights for the contribution of hippocampal abnormalities in these psychiatric disorders.

## Introduction

The Sp4 gene, a member of Sp1 family of transcription factors, recognizes GC-rich elements in the promoter of a variety of genes. These GC-rich sequences are readily identified in the “CpG islands” around the promoters of a variety of genes [1]. Both Sp1 and Sp4 recognize the same DNA binding sequence, and functionally substitute with each other *in vitro* [2]. In contrast to the ubiquitous expression pattern of the *Sp1* gene, the *Sp4* gene is restrictively expressed in nervous system [3], [4]. Our previous studies demonstrated that the complete absence of the *Sp4* gene impaired postnatal development of hippocampal dentate gyrus by reducing cell proliferation, dendritic growth, and dendritic arborization in *Sp4* null mutant mice [5]. Moreover, hypomorphic *Sp4* mutant mice with reduced expression of the *Sp4* gene displayed vacuolization in the hippocampus as well as deficits in sensorimotor gating and memory, putative endophenotypes for several psychiatric disorders including schizophrenia and bipolar disorder [4], [6], [7].

On the other hand, the human SP4 gene has been mapped to chromosome 7p15, where a susceptibility locus was suggested for a broad spectrum of human psychiatric disorders, including schizophrenia [8], [9], [10], panic disorder [11], [12], ADHD [13], autism [14], [15], [16], [17], and bipolar disorder [18], [19], [20]. Of these genome linkage studies, we were particularly interested in the finding that one locus on chromosome 7, at the marker D7S1802, was found to associate with bipolar disorder, which is about 700 kb away from the SP4 gene [18], [21]. Another genome scan also suggested a susceptibility locus for bipolar disorder on chromosome 7p15 [19]. All these psychiatric disorders displayed sensorimotor gating deficit (18). We therefore hypothesized that a susceptibility gene may contribute to the pathogenesis of these different clinical disorders by modulating sensorimotor gating, an endophenotype for several psychiatric disorders. Considering deficient sensorimotor gating in Sp4 hypomorphic mice and human SP4 gene localized in a susceptibility locus for bipolar disorder, we therefore examined whether human SP4 gene may associate with bipolar disorder and schizophrenia. We selected ten single nucleotide polymorphisms (SNPs) encompassing the human SP4 genomic locus, and examined their association with both bipolar disorder and schizophrenia in European and Chinese Han populations, respectively. Significant associations were observed between SP4 gene and bipolar disorder/schizophrenia in all three independent samples. Interestingly, our further studies found that the expression of mouse *Sp4* gene exhibits haploinsufficiency for modulating sensorimotor gating. Moreover, administration of either dopamine D2 antagonists or mood stabilizers can partially reverse the sensorimotor gating deficits.

## Materials and Methods

### UCSD and NIMH families with bipolar disorder

The total sample for analysis included 1872 individuals from 427 Caucasian families, approximately half of which consisted of both parents and one affected offspring with the remaining consisting of both parents and two or more affected offspring. The average family consisted of 4.4 individuals in 2 generations. The families chosen for this study derived from several sources. The National Institute of Mental Health Genetics Initiative for Bipolar Disorder waves 1–4 pedigree collections were collected as part of a consortium and ascertained through a bipolar I proband, and assessed using the Diagnostic Interview for Genetic Studies (DIGS) [22]. Families were also drawn from the University California San Diego consortium including collection sites at UCSD, University of British Columbia, and University of Cincinnati [23]. These families were ascertained through a bipolar I or bipolar II proband and selected for the presence of at least two other mood disordered family members. The Structured Clinical Interview for DSM-III-R (SCID) was used to directly interview subjects for this sample. For each collection, information from the interview, other family informants, and medical records were then reviewed by a panel of clinicians in order to make a final best-estimate diagnosis. DNA of all study subjects was prepared from cultured lymphoblastoid cells.

### Chinese bipolar cases and control sample

506 unrelated bipolar patients (male: 284, female: 222; average of age: 37.8±11.4 years; average age of onset: 26.7±10.6 years) and 507 unrelated controls (male: 287, female: 220; age: 36.4±8.7 years) were recruited for the study. All the subjects were of Han origin from Anhui province. Among the 506 bipolar patients diagnosed using DSM-IV, 419 (82.8%) patients were type I Bipolar. Final diagnosis was made with two independent psychiatrists on the basis of interview and medical records. All participating subjects provided written informed consent according to procedures approved by the Ethic Committee of Bio-X Center, Shanghai Jiao Tong University.

### Chinese trio families with schizophrenia

A total of 325 trio families were collected from several different sites with 126 families from Shanghai, 71 families from Shanxi province, 86 families from Jilin province, and 42 families from Changchun. All subjects were Han Chinese in origin. There were 145 female and 163 male probands with an average age of 24.0±6.6. Clinical diagnoses were made according to DSM-IV, and ascertained by an independent clinician. All the subjects provided written informed consent according to procedures approved by the Ethic Committee of Bio-X Center, Shanghai Jiao Tong University.

### Genotyping

Human SP4 gene encompasses about 90 kb on chromosome 7p15. To conduct a comprehensive association study on Sp4 gene with bipolar disorder in European Caucasian families, we selected ten SNPs which started from the site 5 kb upstream of human SP4 transcription start site and ended at the site 3 kb downstream of its transcription termination. The ten SNPs have good heterozygosity and are representative for LD structures of human SP4 gene in European Caucasian population according to HapMap data. The same set of SNPs was used for replication studies in Chinese bipolar case control sample. A subset of 5 of these SNPs with MAFs >0.10 were genotyped in the Chinese schizophrenia trio families. The primer pairs for the genotyping of individual SNPs were ordered from Applied Biosystems. All SNPs were genotyped in Caucasian and Chinese samples using the TaqMan allele specific assay method (Applied Biosystems) according to the manufacturer's protocols. Concentrations and cycling parameters were optimized to produce clusters of values for heterozygotes and homozygotes separated by >4 standard deviations. The allele frequency of SNP7 (rs12673091) was also confirmed by directly sequencing in Chinese case control sample for bipolar disorder.

### Genetic association analysis

In the Caucasian sample, Haploview v.4.0 [24] was used for an assessment of linkage disequilibrium (LD) within the gene, as well as calculations of allele frequencies and Hardy-Weinberg Equilibrium (HWE), based on unrelated individuals in all samples. The program HAP [25] was used for an evaluation of the block structure of SP4 in the Caucasian bipolar family sample, and the resulting structure was used to inform subsequent haplotype analyses. Haplotypes for unrelated individuals (i.e., parents) in the sample were determined using the imperfect phylogeny phasing method implemented in HAP. Following phasing, HAP partitioned the region into haplotype blocks of correlated SNPs using an algorithm that minimizes the number of tag SNPs [25]. Following block and haplotype identification, the phylogenetic relationships between haplotypes were computed by optimizing a probabilistic model that assumes exponential population growth after a population bottleneck. Under this assumption, any variants after the bottleneck occur in haplotypes that are also present in the modern population. Haplotypes that have no intermediates between them (i.e., that vary by ≥2 SNPs) are assumed to be before the bottleneck and are therefore “ancestral” haplotypes. “Common” haplotypes are defined as being a single mutation or recombination event away from the ancestral haplotypes as such events are likely to have happened after a major population bottleneck. The algorithm identifies “recent” haplotypes by considering rare (<5%) haplotypes and explaining them with either a single gene conversion, mutation, or recombination event from the “common” haplotypes. Haploview [24] was then used to perform the parenTDT test using single SNPs and haplotypes. In addition to providing protection against population stratification, the parenTDT can also add considerable power to family-based association analyses if the parents are measured on the phenotype, as most of ours are, and are discordant [26]. For the haplotype analyses, counts were obtained by summing the fractional likelihoods of each individual for each haplotype. Permutations tests were performed to correct for multiple comparisons. UNPHASED [27] was used for genotypic association analyses. All association analyses were performed under a definition of bipolar disorder that included bipolar I and II, as well as schizoaffective disorder, bipolar-type.

Haploview v.4.0 [24] was used to calculate allele frequencies, Hardy-Weinberg Equilibrium (HWE), and p value of both single SNPs and haplotypes in Chinese BP case control samples. The LD block structure was calculated using the four gamete rule, a variant on the algorithm described by Wang et al [28]. For each marker pair, the population frequencies of the 4 possible two-marker haplotypes were computed. If all 4 are observed with at least frequency 0.01, a recombination is deemed to have taken place. Blocks are formed by consecutive markers where only 3 gametes are observed. The 1% cutoff can be edited to make the definition more or less stringent.

The family-based association analyses of the single SNPs and haplotypes in the Chinese schizophrenia trio families were performed using the TDTPHASE module implemented in the UNPHASED program [29]. An E-M algorithm was used to estimate missing genotypes and excessively rare haplotypes were removed from the analyses.

### Sp4 hypomorphic mice

The *Sp4* hypomorphic mice were generated as described [4], and maintained in both S129 and Black Swiss, respectively. The *Sp4* test cohort was generated by breeding the heterozygous *Sp4* mice between the two genetic backgrounds. Therefore, all the test mice will have the same genetic mixed S129/Black Swiss background as the F1 generation. PCR was used for *Sp4* mouse genotyping as previously described [4]. Mice were housed in a climate-controlled animal colony with a reversed day/night cycle. Food (Harlan Teklab, Madison, WI) and water were available *ad libitum*, except during behavioral testing. All behavioral testing procedures were approved by the UCSD institutional animal care and use committee prior to the onset of the experiments. Mice were maintained in American Association for Accreditation of Laboratory Animal Care approved animal facilities at the local VA Hospital. This facility meets all Federal and State requirements for animal care.

### Prepulse inhibition session

Startle reactivity was measured using eight startle chambers (SR-LAB, San Diego Instruments, San Diego, CA) with background noise of 65 dB and the various acoustic stimuli. All PPI test sessions consisted of startle trials (PULSE-ALONE), prepulse trials (PREPULSE+PULSE), and no-stimulus trials (NOSTIM). The PULSE-ALONE trial consisted of a 40-ms 120-dB pulse of broad-band noise. PREPULSE+PULSE trials consisted of a 20-ms noise prepulse, 80 ms delay, then a 40-ms 120-dB startle pulse (100 ms onset to onset). The acoustic prepulse intensities were 69, 73, and 81 dB (ie 4, 8, and 16 dB above the 65-dB background noise). The NOSTIM trial consisted of background noise only. The test session began and ended with five presentations of the PULSE-ALONE trial; in between, each acoustic or NOSTIM trial type was presented 10 times in a pseudo-random order. There was an average of 15 s (range: 12–30 s) between trials. A background noise level of 65 dB was presented for a 10-min acclimation period and continued throughout the test session.

### Drugs

Raclopride (3 mg/kg, Sigma, St. Louis, MO, USA) was dissolved in distilled water and injected intraperitoneally at a volume of 5 ml/kg 10 min before the start of the test session. Lithium chloride anhydrous (85 mg/kg, Sigma-Aldrich) was dissolved in saline and administered i.p. at a volume of 5 ml/kg 60 min before the start of the test session. The sodium salt of valproate (100 mg/kg, Sigma-Aldrich) was dissolved in saline and administered i.p. at a volume of 5 ml/kg 10 min before the start of the test session. Carbamazepine (25 mg/kg, Sigma-Aldrich) was suspended in saline containing 5% Tween 80 and maintained at 60°C in the dark, and administered i.p. at a volume of 10 ml/kg 40 min before the start of the test session.

### Data Analysis

The amount of PPI was calculated as a percentage score for each acoustic prepulse trial type: % PPI = 100−{[(startle response for PREPULSE+PULSE)/(startle response for PULSE-ALONE)]×100}. For statistical analyses, repeated measures analysis of variance (ANOVA) with genotype as a between subject factor and drug treatment, block and prepulse intensity as within subjects factors was performed on the %PPI data. The same analysis was also performed on the acoustic startle data (PULSE alone trials). Where appropriate, post hoc analyses were carried out using Newman-Keuls or Tukey's test. Alpha level was set to 0.05. All statistical analyses were carried out using the BMDP statistical software (Statistical Solutions Inc., Saugus, MA).

## Results

### European Caucasian Families for Bipolar Disorder

**Figure 1:**
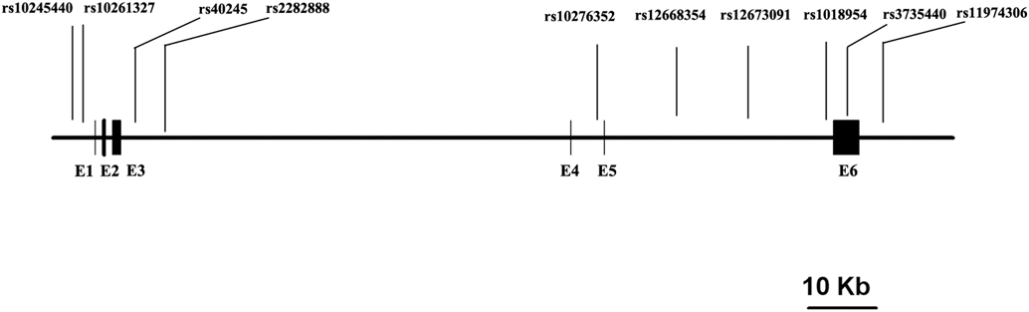
Human SP4 genomic structure. Ten SNPs were selected from the site 5 kb upstream of human SP4 transcription start site and to the site 3 kb downstream of its transcription termination. All ten SNPs have good heterozygosity in European Caucasian population according to HapMap data.

Ten SNPs spanning the human SP4 locus were selected from both public (Genome Browser, UCSC) and proprietary (Celera) SNP databases (Figure 1). Following genotyping, HAP [25] was used for an evaluation of the block structure of SP4, and Haploview [24] was used for an assessment of linkage disequilibrium (LD) and association. None of the ten SNPs were found to deviate from HWE (p>0.05) in this sample, and all were quite common with minor allele frequencies (MAFs) >0.20.

Table 1 describes the results of the single-SNP association analyses using the parenTDT test. SNP 7 (rs12673091) showed the most significant association in the allelewise analyses with a p value of 0.0005 (permutation p = 0.0007) and an overtransmission of the major (“A”) allele. This SNP also revealed a genotypic effect with an overall p value of 0.009. The p values for the A/A and A/G genotypes were 0.004 and 0.031, respectively, consistent with the significant overtransmission of the “A” allele in the allelewise analyses. Three other SNPs (SNP 6, 9, and 10) were nominally significant, possibly due to their correlations with SNP 7. Interestingly, one of these SNPs, SNP 9 (rs3735440), is located within a potential microRNA (miR-145) binding site in the 3′ untranslated region of human SP4 gene, and displayed nominally significant (p = 0.019) overtransmission of the “C” allele.

**Table 1:** Association analysis of SP4 gene with both bipolar disorder and schizophrenia.

SNP #	SNP ID	Caucasian Families for Bipolar Disorder						Chinese Case-Controls for Bipolar Disorder						Chinese Trios for Schizophrenia				
		Alleles*	MAF	Risk	T∶U	Chisq	P	Alleles*	MAF	Risk	Case, Control	Chisq	P	MAF	Risk	T∶U	Chisq	P
1	rs10245440	C/A	0.24	A	195∶192	0.2	0.647	A/C	0.46	C	0.47, 0.44	1.74	0.187	0.45	C	139∶138	0.00	0.955
2	rs10261327	C/T	0.46	C	246∶227	0.8	0.379	T/C	0.49	C	0.49, 0.49	0.00	0.953	0.47	C	139∶137	0.01	0.908
3	rs40245	T/A	0.38	A	269∶251	1.2	0.273	**T/A**	**0.06**	**A**	**0.08, 0.05**	**6.76**	**0.009**					
4	rs2282888	A/G	0.39	G	265∶231	3.3	0.07	A/G	0.09	G	0.10, 0.08	3.36	0.067					
5	rs10276352	A/G	0.44	G	294∶268	2.7	0.102	G/A	0.28	G	0.74, 0.71	2.79	0.095	0.28	G	132∶122	0.40	0.526
**6**	**rs12668354**	**A/C**	**0.33**	**C**	**259∶216**	**5.2**	**0.022**	A/C	**0.09**	C	0.09, 0.08	1.94	0.163					
**7**	**rs12673091**	**A/G**	**0.28**	**A**	**250∶186**	**12**	**0.0005**	**G/A**	**0.13**	**A**	**0.15, 0.10**	**9.47**	**0.002**	**0.11**	**A**	**76∶48**	**6.32**	**0.012**
8	rs1018954	T/A	0.42	A	279∶267	0.3	0.592	**T/A**	**0.04**	**A**	**0.05, 0.02**	**10.22**	**0.001**	0.05	T	39∶27	2.31	0.129
**9**	**rs3735440**	**T/C**	**0.34**	**C**	**274∶225**	**5.5**	**0.019**	**T/C**	**0.08**	**C**	**0.10, 0.07**	**4.79**	**0.029**					
**10**	**rs11974306**	**A/T**	**0.5**	**T**	**298∶251**	**5.6**	**0.018**											

* Alleles for each SNP are presented as major/minor, respectively.

Key: MAF = minor allele frequency; Risk = risk allele; T∶U = transmitted to untransmitted ratio.

**Figure 2:**
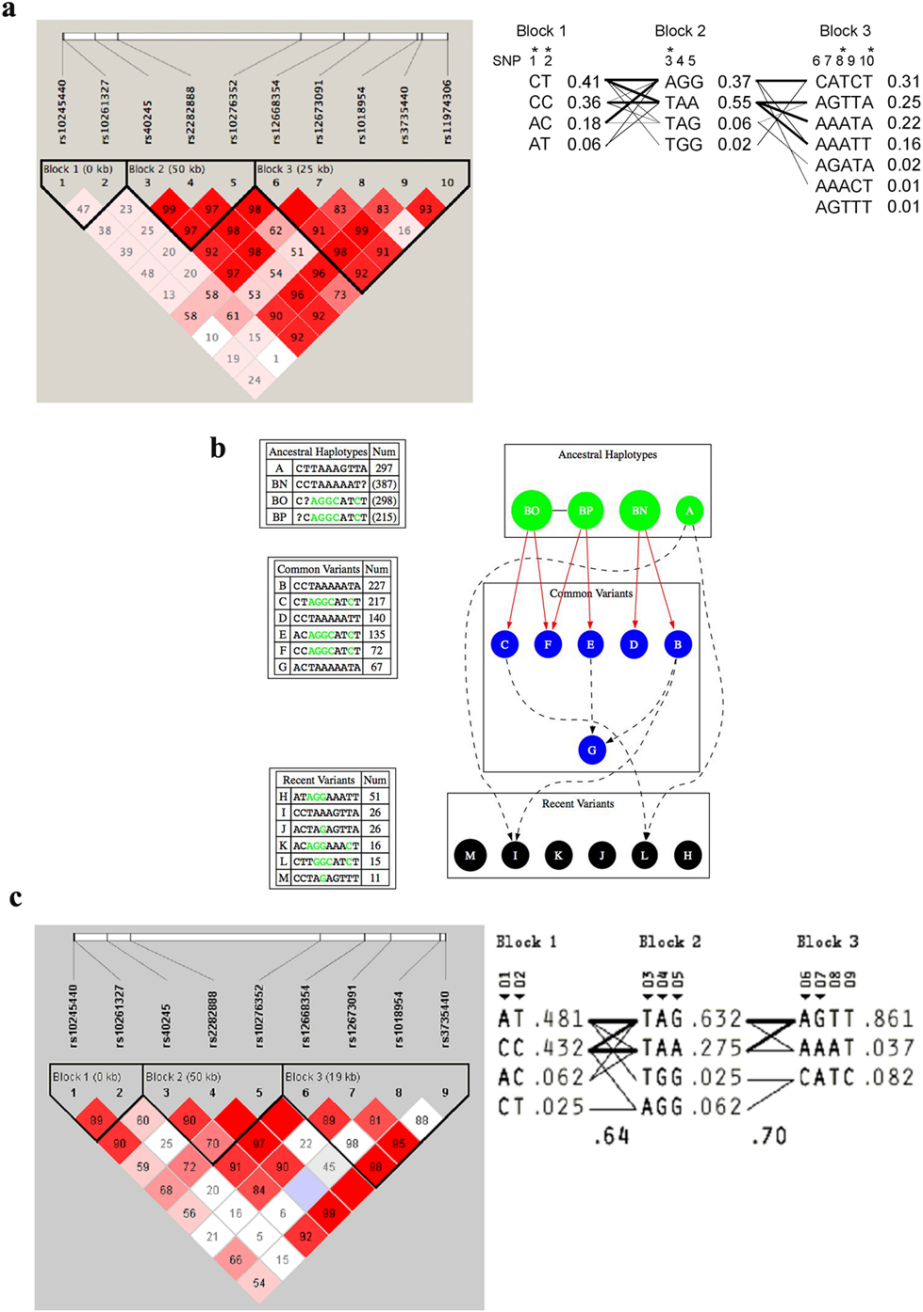
The LD pattern of human SP4 gene. (a) Detailed LD structure of SP4 gene in the UCSD/NIMH sample, along with the designation of the three haplotype blocks, the haplotypes within, and the relationships of haplotypes between blocks. Measures of the strengths of the LD are indicated within each block, which are color-coded with red being high LD and white being low LD. An (*) indicates a tagging SNP. Heavy solid lines indicate a frequency >0.10, whereas solid lines indicate a frequency >0.01. The frequencies of each haplotype are indicated to the right. (b) Predicted phylogeny of the SP4 gene. Solid lines indicate single mutation events in a lineage, and dashed lines indicate recombination events in a lineage (there are necessarily two sources for each resulting daughter haplotype). “Ancestral”, “common,” and “recent” haplotypes are defined in the Methods section. A “?” in an ancestral haplotype represents the precursor to a mutation, which allows for the presentation of the information that the two haplotypes are related, knowing where the mutation occurred but not which haplotype is the parent. (c) Details of the LD and haplotype structure of the SP4 gene in the Chinese BPAD case-control sample.

An assessment of LD across the SP4 gene revealed three LD blocks with a high degree of LD observed between SNPs 6–10, all of which derive from block 3 and four of which displayed at least marginal evidence for association (Figure 2a). We therefore performed haplotypic association analyses on all haplotypes from block 3 with frequencies >0.01. The results of these analyses are shown in Table 2. The global p value was 0.004 with one haplotype (AGTTA) in particular revealing a significant undertransmission and a p value of 0.0004 (permutation p = 0.001). This haplotype contains the “G” allele of SNP 7, which was the previously undertransmitted allele in the single SNP analyses. Another haplotype (CATCT) containing the previously overtransmitted “A” allele was marginally significant as well. Phylogenic analyses of haplotypes composed of all ten SNPs, as shown in Figure 2b, reveal that this overtransmitted block 3 haplotype derives from two related ancestral haplotypes (haplotypes BO and BP) that form a haplotype cluster of several related common haplotypes. On the other hand, the significantly undertransmitted block 3 haplotype (AGTTA) comprises a rather distinct ancestral haplotype (haplotype A) that is relatively common but for which there are only a few relatively rare recent haplotype derivatives. Common haplotypes derived from the other ancestral haplotype (haplotype BN) did not show an association with bipolar disorder.

**Table 2:** Haplotype analyses of SP4 association in bipolar samples.

Caucasian Families for Bipolar Disorder					Chinese Case-Controls for Bipolar Disorder				
Block 3 (SNP 6–10)	Freq.	T∶U	Chisq.	P Value*	Block 3 (SNP6–9)	Freq.	Case,Control Freq.	Chisq.	P Value
**CATCT**	**0.31**	**249.3∶215.9**	**5.50**	**0.019**	**CATC**	**0.082**	**0.096, 0.069**	**4.87**	**0.027**
**AGTTA**	**0.25**	**179.9∶229.3**	**12.45**	**0.0004**	**AGTT**	**0.861**	**0.846, 0.877**	**4.22**	**0.040**
AAATA	0.22	195.9∶197.8	0.17	0.684	**AAAT**	**0.037**	**0.046, 0.028**	**4.69**	**0.030**
AAATT	0.16	140.2∶126.2	0.43	0.511					
AGATA	0.02	12.8∶19.1	1.43	0.232					
AGTTT	0.01	15.4∶16.5	0.01	0.907					
AAACT	0.01	19.0∶18.0	0.30	0.585					

* Global p = 0.004.

### Chinese Case-Control Sample for Bipolar Disorder

To provide a replication of these results, the same ten SNPs were genotyped in a sample of 506 Chinese bipolar cases and 507 controls. SNP10 (rs11974306) was found to deviate from HWE in this sample and thus removed from further analyses. These SNPs were generally more rare in the Chinese population with only five of the ten SNPs displaying MAFs >0.10, suggesting population differences between individuals of Caucasian and Chinese ancestry for this gene. Four SNPs differed significantly between cases and controls. SNP7 (rs12673091) displayed a significant association in the Chinese bipolar case-control sample with an overtransmission of the minor (“A”) allele s (p value, 0.002; permutation p value 0.014), thereby replicating the results of our analyses in the Caucasian family sample (see Table 1). Moreover, SNP9 (rs3735440) displayed a moderately significant association (p = 0.029) with an overtransmission of the “C” allele in Chinese population, also replicating our results in the Caucasian family analyses.

Similar to the Caucasian sample, a high degree of LD between SNPs 6–9 was observed in the Chinese sample (Figure 2c). Since three of the four SNPs significantly associated with bipolar disorder in this sample all lie within this LD block (block 3), we performed association analyses of all haplotypes composed of these three SNPs. Two haplotypes CATC (p = 0.027) and AGTT (p = 0.04) displayed significant overtransmission and undertransmission, respectively, in this sample, consistent with the observed associations in the Caucasian families (Table 2).

### Chinese Trio Families for Schizophrenia

It has been suggested that both bipolar disorder and schizophrenia share common genetic risk factors. To explore whether SP4 gene might also be associated with schizophrenia, we conducted genetic studies on a sample of 325 Chinese trio families with schizophrenia. We observed an association between SNP7 (rs12673091) and schizophrenia in the Chinese population (p = 0.012) with a significant overtransmission of the “A” allele (see Table 1). These results are thus consistent with those for SNP7 in both the Caucasian and Chinese bipolar samples.

All together, we observed a significant genetic association between human SP4 gene and bipolar disorder and schizophrenia in all three independent samples. To correlate these positive SNPs with the level of human SP4 gene expression, we extracted total RNA from the lymphoblastoid cells of European Caucasian samples. Unfortunately, we could not detect the expression of human SP4 gene by RT-PCR. The neuronal restrictive expression of SP4 gene prevented our further effort to investigate the effects of the positive SNPs on the level of human SP4 gene expression. However, the expression of human SP1 gene, which functionally substitutes with SP4 gene by recognizing the same DNA binding motif [2] , was found to be reduced in both prefrontal cortex and striatum of postmortem brains of schizophrenia patients [30]. Therefore, reduced expression of SP1 family transcription factors may play an important role in pathophysiology of schizophrenia and related disorders. Consistent with this notion, mice with reduced expression of *Sp4* gene displayed deficient sensorimotor gating and memory.

### Haploinsufficiency for Mouse Sp4 Gene in Modulating Sensorimotor Gating

**Figure 3:**
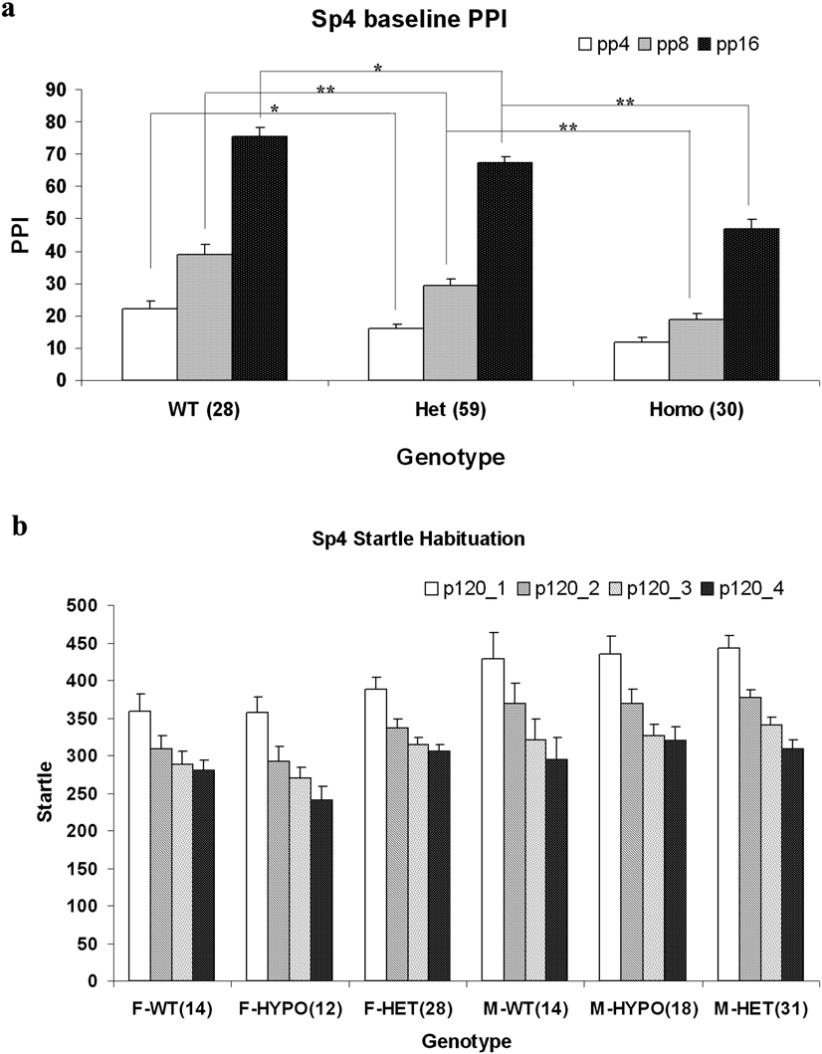
Haploinsufficiency of mouse *Sp4* gene in the modulation of prepulse inhibition. The number of mice for the PPI studies were included in the parentheses after individual genotype (a) Prepulse inhibition at three different prepulse levels. (b) Startle habituation within the PPI session. * p<0.05; ** p<0.01.

Sensorimotor gating, a putative endophenotype for both schizophrenia and bipolar disorder, has been studied extensively in both human patients and animal models [6], [31], [32], [7]. Our previous studies demonstrated that hypomorphic *Sp4* mutant mice, in which expression of the *Sp4* gene is reduced to 2%–5% of the level in wildtype mice, displayed a variety of behavioral abnormalities including deficient sensorimotor gating [4]. To further examine whether the level of *Sp4* gene expression has dosage effects on sensorimotor gating, we conducted prepulse inhibition tests on *Sp4* heterozygous mice as well as their sibling wildtype and homozygous mutant mice. There was a significant *Sp4* gene effect on PPI with p value less than 0.00001 (Figure 3a). *Post hoc* analysis revealed that there were significant differences in PPI between wildtype and heterozygous *Sp4* mice at all three different prepulse levels. The homozygous *Sp4* mutants also displayed significantly reduced PPI relative to heterozygous *Sp4* mice which in turn have less PPI than wildtype siblings, consistent with a gene-dosage effect. No gender effect was observed in the PPI tests. All mice displayed normal startle habituation, with no difference among different genotypes in startle magnitude (Figure 3b). Nevertheless, male mice tend to have higher magnitude of startle than female mice, presumably due to their heavier body weight.

### Improvement of PPI Deficit with Dopamine D2 Antagonist and Mood Stabilizers

**Figure 4:**
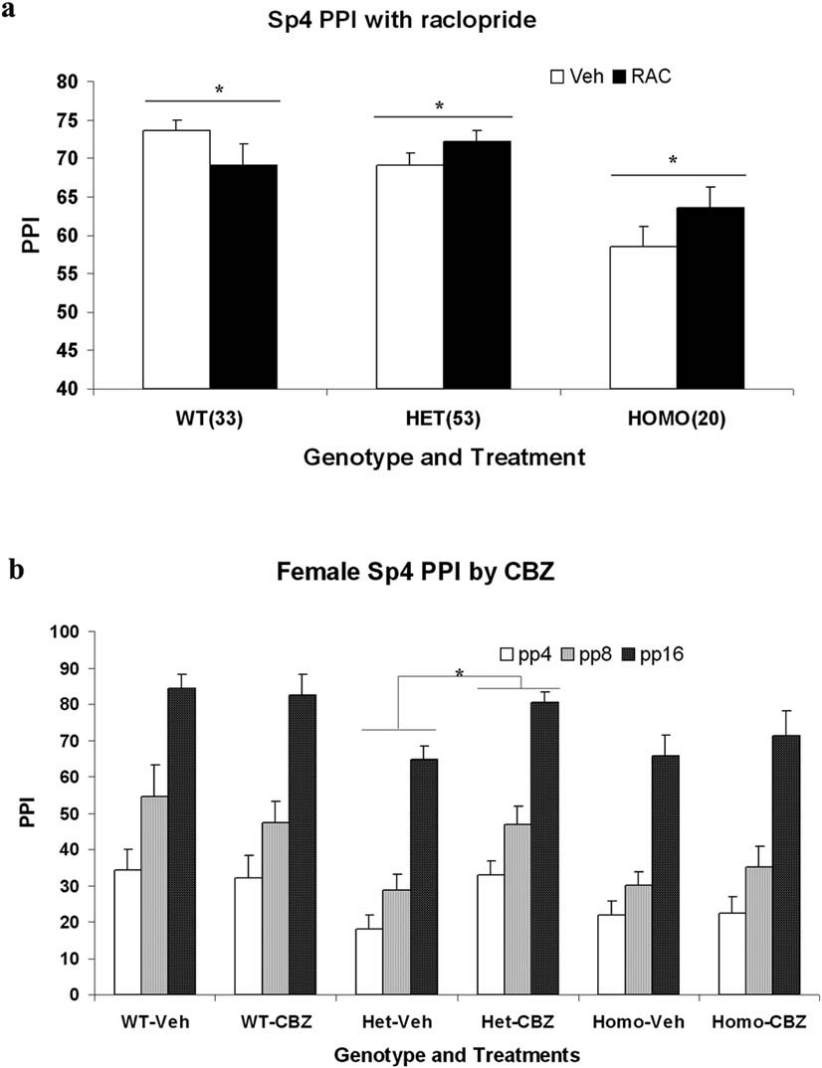
Improvement of sensorimotor gating with dopamine D_2_ antagonist raclopride and carbamazepine in *Sp4* mutant mice. (a) A within subject design was used for the raclopride studies. The average PPI values across three different prepulse intensities were used for comparison. A significant PPI improvement was observed in both Sp4 heterozygous and homozygous mice after the administration of raclopride at the dosage of 3 mg/kg. The numbers of mice for the PPI studies were included in the parentheses after individual genotype (b) A between subject design was used for the carbamazepine studies. There were 7 mice in each group of WT-Veh and WT-CBZ mice, 14 mice in each group of Het-Veh and Het-CBZ mice, 7 mice in each group of Homo-Veh and Homo-CBZ mice. A significant PPI improvement was observed in female *Sp4* heterozygous mice after the administration of carbamazepine at the dosage of 25 mg/kg. * p<0.05.

The importance of dopamine neurotransmission for the pathogenesis of both bipolar disorder and schizophrenia has been suggested by both the clinical efficacy of dopamine D_2_ receptor antagonists in human patients and their central roles in modulating relevant behaviors in animal models. As in rodent models of deficient PPI [7], [33], the administration of antipsychotics significantly improved PPI deficits in schizophrenia patients [34], [35]. To examine whether PPI deficits in both *Sp4* heterozygous and homozygous mutant mice can be improved by administration of dopamine D_2_ receptor antagonists, we conducted pharmacological reversal of the PPI deficits with the administration of raclopride. Besides a significant gene effect, a strong gene X raclopride effect on PPI was observed (gene X drug interaction *p* value, 0.0029). No significant effect was observed in the interactions between gene, raclopride and prepulse intensity. Therefore, we averaged the prepulse inhibition values across three different prepulse levels in both vehicle and drug groups. *Post hoc* analyses were conducted to compare the average PPI between vehicle and drug groups in three different genotypes. A significant improvement of PPI with raclopride was observed in both *Sp4* heterozygous and homozygous mice (Figure 4a). Surprisingly, the administration of raclopride decreased PPI in wildtype mice, indicating possible drug effects on D_2_ autoreceptors in the regulation of dopamine release [36], [37]. The observed PPI improvement in both *Sp4* heterozygous and homozygous mice could therefore be underestimated because of the PPI reduction caused by D_2_ autoreceptor inhibition.

Mood stabilizers have been used for the treatment of bipolar disorder. To study whether mood stabilizers can improve the PPI deficits, lithium chloride was administered acutely to reverse the PPI deficits in both *Sp4* heterozygous and homozygous mice. Neither lithium nor gene X lithium effect on PPI was observed (Figure S1). In contrast, we observed that acute treatment with the mood stabilizer valproate improved PPI across all three different genotypes. However, the gene X valproate effect was not statistically significant due to the PPI increase in wildtype mice (Figure S1). Carbamazepine, an anticonvulsant used as a mood stabilizer, also increased PPI across all three genotypes at a dosage of 50 mg/kg. To minimize nonspecific drug effects, we decreased the dosage to 25 mg/kg. Surprisingly, we observed significant improvement of PPI only in female heterozygous mice (Figure 4b). Both wildtype and homozygous female mice did not respond to the administration of carbamazepine at this dosage. To confirm the observation, we examined the effects of carbamazepine on PPI in a separate *Sp4* mouse cohort. We again observed that the reduced dosage of carbamazepine improved PPI only in female heterozygous *Sp4* mice (data not shown). The differential response between *Sp4* heterozygous and homozygous female mice may indicate the role of *Sp4* function in the mediation of carbamazepine drug effect. The failure of PPI improvement in male heterozygous mice was unexpected, however, the gender effect was also observed in their differential startle response to carbamazepine (data not shown) [38].

## Discussion

Our previous studies demonstrated a central role for the *Sp4* gene in hippocampal development and modulation of a variety of behaviors relevant to human psychiatric disorders [4], [5]. The findings prompted us to examine whether human SP4 gene may associate with psychiatric disorders. After conducting genetic association studies, we observed a significant association between the human SP4 gene and bipolar disorder and schizophrenia in all three independent samples. It is especially striking that we saw association of the same alleles and haplotypes in both Caucasian and Chinese populations. Excessive dopamine transmission has been proposed in the pathophysiology of both bipolar disorder and schizophrenia; we therefore examined whether similar disruption of dopamine neurotransmission occurred in hypomorphic *Sp4* mice. As expected, our pharmacological studies suggested that both dopamine D_2_ receptor antagonists and mood stabilizers partially reversed the PPI deficits in *Sp4* mutant mice.

**Figure 5:**
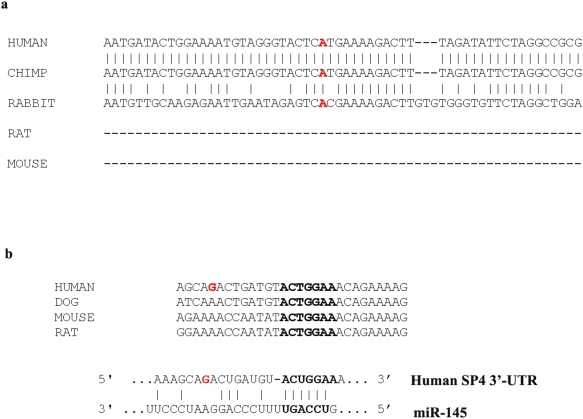
Conservation of sequence polymorphism. Sequence conservation of the SNP 7 (a) and 9 (b) between different species.

Psychiatric disorders are likely caused by multiple susceptibility genes, each with small effects in increasing the risk of illness [39]. Human population heterogeneity constitutes an enormous challenge; few susceptibility genes have so far been confirmed consistently for psychiatric disorders. We conducted two of our three association analyses in nuclear families, which are more robust to the effects of population substructure. The associations were further verified in the Chinese case-control sample, which complement the design of family studies. Significant overtransmission of the “A” allele from SNP7 (rs12673091) was observed in both bipolar and schizophrenia patients in all three independent samples. The “A” allele of SNP7 (rs12673091) was localized in the fifth intron of human SP4 gene and is conserved in both chimpanzee and rabbit, but not in mouse and rat (Figure 5a). SNP9 (rs3735440) also displayed significant association with bipolar disorder in both European Caucasian families and Chinese case-control samples. Interestingly, the overtransmitted “C” allele was localized in the putative binding site of microRNA (miR-145), which recognizes a seed sequence eight nucleotides downstream of the SNP9, in the 3′ untranslated region of the human SP4 gene (Figure 5b). However, both “T/C” alleles cannot base-pair with the corresponding sequence of miR-145. It has been shown that unpaired sequence in the microRNA binding site could also play an important role in the regulation of interactions between microRNA and its target mRNA [40], [41]. In fact, the “T” allele of the SNP9 was conserved from mouse to human (Figure 5b). Therefore, the “C” allele of the SNP9 merits further studies as a potential functional mutation.

In addition to the consistency of single SNP associations in two different ethnic populations, we also observed that similar haplotypes significantly associated with bipolar disorder. In the Caucasian families, the haplotype (AGTTA) from SNP 6–10 (rs12668354, rs12673091, rs1018954, rs3735440, and rs11974306) was undertransmitted with a p value of 0.0004. Interestingly, a version of this haplotype (AGTT) from SNP 6–9 (rs12668354, rs12673091, rs1018954, rs3735440) was also undertransmitted with p value of 0.0399 in Chinese bipolar patients. Similarly, haplotypes CATCT and CATC were significantly overtransmitted in the Caucasian and Chinese populations, respectively. Taken together, these data provide strong support for the hypothesis that the SP4 gene functions as a susceptibility gene for bipolar disorder and possibly for schizophrenia as well, since the two disorders have overlapping genetic components [42], [43]. Unfortunately, the neuronal restrictive expression of SP4 gene prevented our further effort to study the association between these positive SNPs/haplotypes and the level of human SP4 gene expression. However, decreased expression of human SP1 gene, functionally redundant with its family member SP4 gene, was reported in the prefrontal cortex and striatum of postmortem brains of schizophrenia patients [30]. The reduced expression of SP1 family transcription factors may result in aberrant expression of many downstream target genes which contribute to the pathophysiology of schizophrenia and related disorders. Consistent with the reduced expression of SP1 gene in schizophrenia, reduced expression of mouse *Sp4* gene resulted in both hippocampal abnormalities and deficient sensorimotor gating, two putative endophenotypes for schizophrenia and related psychiatric disorders. In this study, we found that the mouse *Sp4* gene exhibits haploinsufficiency in the modulation of sensorimotor gating. This finding may be particularly relevant to consideration of SP4 as a candidate susceptibility gene for psychiatric disorders, as considerable evidence suggests that hypomorphic alleles, instead of loss of function, of multiple genes contribute to the pathogenesis of psychiatric disorders [44].

Excessive dopamine neurotransmission has been demonstrated in both bipolar disorder and schizophrenia. Most clinically effective antipsychotics are dopamine D_2_ receptor antagonists. The administration of raclopride, a dopamine D_2_ antagonist, significantly improved sensorimotor gating in both *Sp4* heterozygous and homozygous mice but not in wildtype mice, suggesting that dopamine neurotransmission may be altered in *Sp4* mutant mice as it is in human psychiatric disorders. Considering significant genetic association between SP4 gene and bipolar disorder, we also examined the effects of different mood stabilizers on the reversal of PPI deficits in mutant mice. The mood stabilizer valproate improved PPI in both wildtype and Sp4 mutant mice. Acutely administrated carbamazepine also significantly reversed the PPI deficit in female heterozygous *Sp4* mice. Although we did not observe effects of acutely administered lithium on PPI improvement, chronic administration of lithium may be necessary for the reversal of PPI deficits. Taken together, the pharmacological studies on *Sp4* hypomorphic mice provided additional support for SP4 gene as a susceptibility gene for bipolar disorder and schizophrenia. In the future, it will be interesting to study whether similar pharmacogenetic interactions in hypomorphic *Sp4* mice may be conserved in human. Such studies would be valuable for not only the cross-species validation of the Sp4 mouse model but also the development of gene-specific diagnosis and treatment.

Our human genetic association studies provide direct evidence for the human SP4 gene as a susceptibility gene for both bipolar disorder and schizophrenia. However, the functional mutations in human SP4 remain to be studied. On the other hand, the elucidation of *Sp4* molecular pathways in the *Sp4* hypomorphic mouse model will provide novel insights in our understanding of neural circuitry in the regulation of sensorimotor gating and other behaviors relevant to human psychiatric disorders.

## Supporting Information

Figure S1 (9.62 MB TIF)
