# Supplementary figures and images for: Transcription Factor SP4 Is a Susceptibility Gene for Bipolar Disorder
Source: PLoS One. 2009 Apr 9;4(4):e5196. doi: 10.1371/journal.pone.0005196 (PMC2674320; doi:10.1371/journal.pone.0005196)

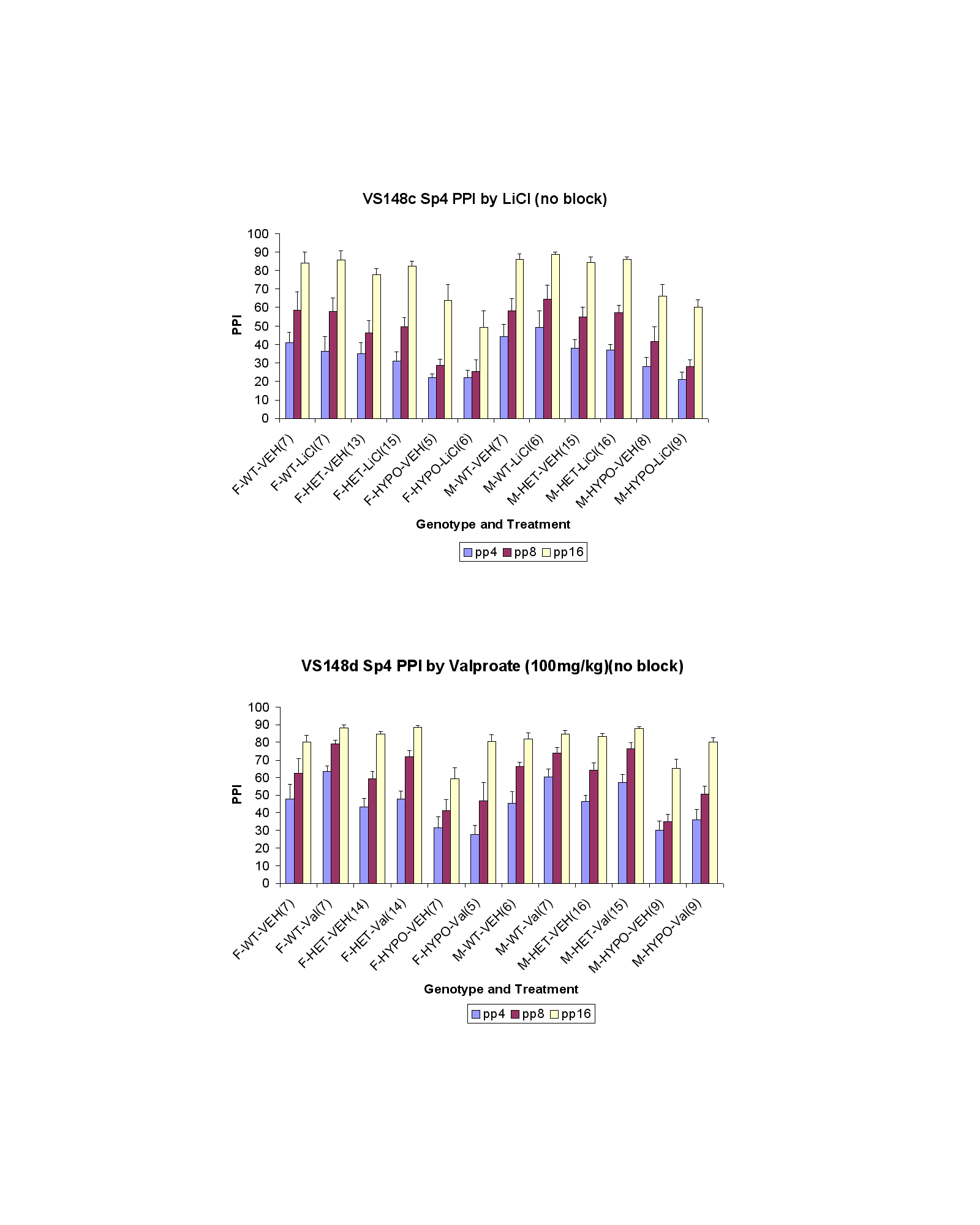

Supplement: Figure S1 — (9.62 MB TIF) [file pone.0005196.s001.tif]
